# ﻿Molecular phylogeny suggests synonymy of *Sandaliabridgesi* Lorenz, 2009 with *S.triticea* (Lamarck, 1810) (Gastropoda, Ovulidae)

**DOI:** 10.3897/zookeys.1096.79402

**Published:** 2022-04-18

**Authors:** Qiong Wu, BingPeng Xing, Mao Lin, GuangCheng Chen, ChunGuang Wang

**Affiliations:** 1 Third Institute of Oceanography, Ministry of Natural Resources, PRC. 178#, Daxue Road, Siming District, Xiamen, Fujian, 361005, China Third Institute of Oceanography, Ministry of Natural Resources XiaMen China; 2 Observation and Research Station of Coastal Wetland Ecosystem in Beibu Gulf, Ministry of Natural Resources, Beihai, 536015, China Observation and Research Station of Coastal Wetland Ecosystem in Beibu Gulf, Ministry of Natural Resources Beihai China

**Keywords:** 16S, COI, DNA sequencing, ITS, molecular phylogeny, taxonomy

## Abstract

The Ovulidae (Gastropoda, Cypraeoidea) is a family of small to medium Mollusca in the order Littorinimorpha, and *Sandalia* is a very small genus containing only three extant species. In the present study, 132 specimens of Ovulidae were collected, belonging to seven genera and nine species, including 54 *Sandaliabridgesi* and three *Sandaliatriticea* individuals. The cytochrome c oxidase I gene, 16S rRNA, and ITS1-5.8S-ITS2 sequences were obtained from all specimens and compared with sequences downloaded from GenBank to calculate genetic distances and construct phylogenetic trees. The sequences of *S.bridgesi* and *S.triticea* exhibited a high degree of similarity, and *S.bridgesi* does not form a separate clade, supporting the proposal that *S.bridgesi* should be synonymised with *S.triticea*.

## ﻿Introduction

The family Ovulidae is a group of small and medium sized molluscs distributed in widely tropical and subtropical seas. *Sandalia* Cate, 1973 is a genus belonging to this family, and its known distribution is Korea, Japan (type locality), New Caledonia, and eastern Australia. Shells are mainly characterised by having a pointed adapical terminal beak, peculiarly curving outer lips, and a shoe-like ventral appearance (Cate 1973). According to data from the World Register of Marine Species (WoRMS, https://www.marinespecies.org) and Worldwide Mollusc Species Data Base (WMSDB, https://www.bagniliggia.it/WMSD/WMSDhome.htm), only three extant *Sandalia* species have been described, namely *S.bridgesi* Lorenz, 2009, *S.meyeriana* (Cate, 1973), and *S.triticea* (Lamarck, 1810). All three species are distributed in the West Pacific region: the type localities are Taiwan Strait, Japan, and New Caledonia, respectively.

Recent collections of 132 specimens of ovulid from Chinese coasts prompted an investigation into the identities of the species of *Sandalia* based on 57 fresh specimens.

*Sandaliabridgesi* differs from its congeners by the obvious and striking transparency of the dorsum in contrast to the calloused labrum and terminals. As described by Lorenz (2009), *S.bridgesi* and *S.triticea* are very similar, with the main differences being as follows: *S.triticea* has lower transparency and usually possesses a red or purple shell and pale-coloured callosities. The middle portion of the dorsal side is normally pale, and the shell is roughly pear-shaped. Under ultraviolet (UV) light, yellow fluorescence can be seen in the terminal collars, callosities, dorsal mid-portion, and sometimes in the entire shell. In contrast, *S.bridgesi* has a relatively uniformly coloured dorsal portion and a wider anterior part and only exhibits fluorescence in a small area at either end of the shell under UV light.

DNA barcoding, which involves using a short DNA sequence for species classification, was used as a tool for species identification and received widespread attention 15 years ago (Meier et al. 2006). This technology breaks through the over-reliance on the personal abilities and experiences of taxonomists in traditional morphological classification and enables the informatisation and standardisation of species identification. In the present study, we sequenced the cytochrome c oxidase subunit I (COI) gene, 16S rRNA, and the ITS1-5.8S-ITS2 (ITS) region for the construction of the phylogenetic trees to elucidate the relationship between *S.bridgesi* and *S.triticea*. We obtained sequence data of the ITS region of Ovulidae for the first time.

## ﻿Materials and methods

### ﻿Specimen collection

**Figure 1.**
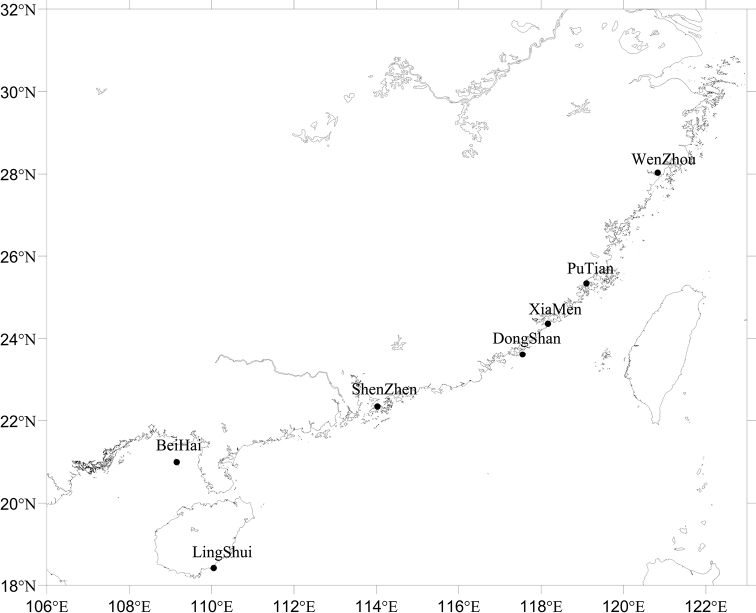
Sample collection locations along the Pacific coast of China.

We collected 132 specimens of Ovulidae from depths of 0–6 m during low spring tides in Hainan, Guangxi, Guangdong, Fujian, and Zhejiang provinces between July 2020 and September 2021. Detailed information of the collected specimens is shown in Fig. 1, Suppl. material 1: Table S1. We have tried but failed to obtain specimens from museums abroad. We also attempted to use the Jiang et al. (2019) method to extract DNA from shells of the Institute of Oceanology (CAS). As the method requires a minimum of 100 mg of sample and our samples were too small, our attempt failed.

**Figure 2.**
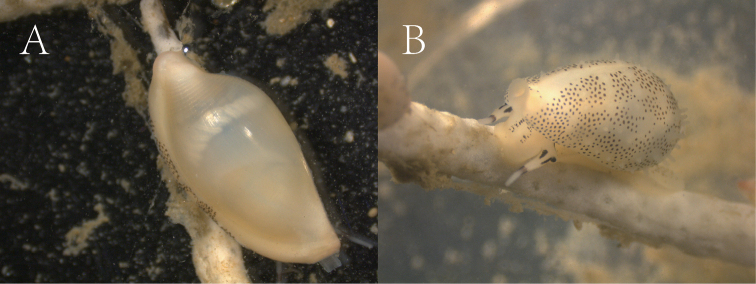
Living animals of *Sandaliabridgesi* Lorenz, 2009 **A** sample 20201117H19 **B** sample 20201117H6.

**Figure 3.**
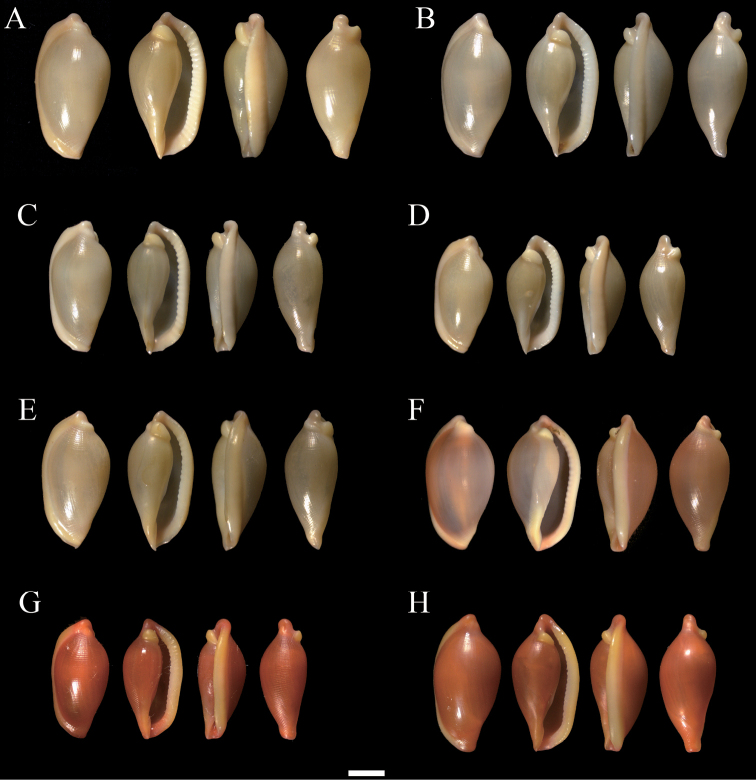
Dorsal, ventral, and lateral views of shells of *Sandalia***A***S.bridgesi* Lorenz, 2009 (20200722H1) **B***S.bridgesi* (20200722H5) **C***S.bridgesi* (20200722H6) **D***S.bridgesi* (20200722H9) **E***S.bridgesi* (20200722H10) **F***S.triticea* (Lamarck, 1810) (20210626H14) **G***S.triticea* (20200722T1) **H***S.triticea* (20200722T2). Scale bar: 2 mm.

All specimens were morphologically identified by WQ, Fan Shihao, and Han Yida in accordance with the identification keys published by Ma (1997), Zhang and Wei (2011), Lorenz and Fehse (2009), Lorenz (2009), and Hardy (2020a), as well as in Worldwide Mollusc Species Data Base (https://www.bagniliggia.it/WMSD/WMSDhome.htm). The specimens were observed and photographed alive (Fig. 2) and from the dorsal, ventral, and lateral sides under a Leica S9D stereomicroscope (Fig. 3, Suppl. material 7: Fig. S1). Specimens were preserved in 95% alcohol.

### ﻿DNA extraction and sequencing

Amplification was performed on three gene regions for each specimen, namely the mitochondrial markers 16S rRNA and COI, and the nuclear ribosomal internal transcribed spacer (ITS) region.

DNA was extracted from each muscle tissue using the DNeasy Blood & Tissue Kit (QIAGEN, China) following the corresponding protocol for animal tissues. The nucleic acid concentration in the DNA extracts was measured using BioDrop (BioDrop, UK). Due to the presence of inhibitors in the specimen tissues, all DNA extracts were diluted 50–500 fold before PCR amplification (Reijnen and van der Meij 2017, 2019). Our experimental results indicated that the appropriate concentration for the diluted DNA extracts was approximately 0.2 µg/mL.

Each PCR had a reaction volume of 50 µL and contained the following: 25 µL PCR mixture [Taq plus Master Mix II (Dye Plus)], 2 µL of each primer (10 µM), 5 µL (diluted) DNA extract, and 16 µL extra pure water. The details of the PCR performed for the three gene regions are given in Table 1. Not all markers were successfully amplified for all specimens, but the successfully amplified COI and 16S rRNA samples were sent to Sangon Biotech Co., Ltd (Shanghai, China) for PCR cleaning and sequencing.

**Table 1.** Details of gene regions and associated primer pairs used in the study.

Gene region	Fragment size (bp)	Primers	Annealing temperature	Reference
COI	~680	Lco1490/Hco2198	45 °C, +0.5 °C /cycle,15 cycle, 49 °C, 20 cycle	Vrijenhoek (1994)
16S	~550	16SAR/16SBR	52 °C	Reijnen and van der Meij (2019)
ITS1-5.8S-ITS2	~1200	GastF/GastR	56 °C	Hoy and Rodriguez (2013)

The quality of the direct sequences obtained for the ITS region was insufficient because of intra-individual variation, secondary structures, and simple sequence repeats (SSRs). Thus, the PCR products were sent to Sangon Biotech Co., Ltd (Shanghai, China) for TA cloning and sequencing. DNA fragments were cloned into *Escherichiacoli* cells using the pESI-T Vector System. For each individual, 3–5 clones were sequenced, and the most common sequence of these positive clones was used in the alignment and ITS data treatments. The sequences have been submitted to GenBank (http://www.ncbi.nlm.nih.gov) and the accession data is provided in Suppl. material 1: Table S1.

### ﻿DNA data processing and molecular analyses

Joining and alignment of the sequences and trimming of ends with low signal strength were performed using DNAMAN v. 9 (Lynnon Biosoft, Canada) and SeqMan v. 7.1.0 (DNAStar, USA). Multiple sequences were aligned with MAFFT (Katoh and Standley 2013) using ‘auto’ strategy. One sequence obtained from GenBank was considered as an outgroup (Suppl. material 3: Table S3). The best-fit evolutionary models were selected based on Bayesian Information Criterion (BIC) by using ModelFinder (Kalyaanamoorthy et al. 2017). Bayesian-inference phylogenies were inferred using MrBayes v. 3.2.6 (Ronquist et al. 2012) (2 parallel runs, 2000000 generations), in which the initial 25% of sampled data were discarded as burn-in. Maximum-likelihood (ML) phylogenies were inferred using IQ-TREE (Nguyen et al. 2015) for 1000 standard bootstraps, as well as the Shimodaira-Hasegawa-like approximate likelihood-ratio test (Guindon et al. 2010). The phylogenetic trees were viewed and edited using iTOL (available at https://itol.embl.de/) following Letunic and Bork (2021).

Evolutionary divergence analyses were conducted in MEGA v. 11(Kumar et al. 2018) and using the Jukes-Cantor model (Jukes and Cantor 1969) (Suppl. materials 4–6: Tables S4–S6). The sequence obtained from GenBank has also been added to the analysis (Suppl. material 3: Table S3).

## ﻿Results

### ﻿Morphological data

Based on the photographs and descriptions provided by Lorenz (2009), Lorenz and Fehse (2009), and Hardy (2020a), the 132 specimens were identified as belonging to nine species in seven genera. Fifty-seven of the specimens were *Sandalia* species, among which 54 were identified as *S.bridgesi* and three were identified as *S.triticea* based on differences in shell transparency, external appearance, and colour. Suppl. material 1: Table S1 shows the information and identification outcomes of the collected specimens. Suppl. material 2: Table S2 shows the length/width (L/W) ratio of shells of *S.meyeriana*, *S.bridgesi*, and *S.triticea*.

### ﻿Molecular data

In total, 122 COI sequences were successfully amplified. After editing, the consensus length of all barcode sequences was 615 bp, and no stop codons, insertions, or deletions were observed in any of the sequences. The sequences were aligned with the 16 COI sequences obtained from GenBank, with detailed information of downloaded sequences provided in Suppl. material 3: Table S3. Phylogenetic trees were constructed using Bayesian and ML methods, and the root location was confirmed by selecting the COI sequence of *Mauritiaarabica* as the outgroup (Suppl. material 8: Fig. S2). The best models of the phylogenetic trees are provided in Tables 2, 3. As the results from the two different phylogenetic reconstructions were congruent at the species level, only the ML tree is illustrated in this paper (Suppl. material 8: Fig. S2).

**Table 2.** The best evolutionary models of ML phylogenomic tree.

Gene region	The best fit models	Reference
COI	HKY+I+G4+F	Hasegawa et al. (1985)
16S	TPM3+G4+F	Kimura (1981)
ITS1-5.8S-ITS2	HKY+F+G4	Hasegawa et al. (1985)

The greatest and smallest genetic distances between *S.bridgesi* and *S.triticea* among our specimens were 0.0215% (*Sandaliabridgesi* (MW410840) and *Sandaliatriticea* (MW410844)) and 0% (*Sandaliabridgesi* (MW410824) and *Sandaliarhodia* (= *triticea*; MG450349); *Sandaliabridgesi* (OL674267) and *Sandaliarhodia* (= *triticea*; MG450349), respectively. The smallest and greatest interspecific genetic distances among specimens other than *S.bridgesi* and *S.triticea* were 0.1220% (*Primovulaformosa* (OL674268) and *Crenavolvatraillii* (OL471931)), and 0.2663% (*Phenacovolva* sp. (OL471933) and *Crenavolvatraillii* (OL471920)), respectively (for more details see Suppl. material 4: Table S4).

**Table 3.** The best models of Bayesian phylogenomic tree.

Gene region	The best fit models	Reference
COI	HKY+I+G+F	Hasegawa et al. (1985)
16S	HKY+G+F	Hasegawa et al. (1985)
ITS1-5.8S-ITS2	HKY+F+G4	Hasegawa et al. (1985)

One hundred 16S rRNA sequences with lengths of approximately 520 bp were successfully amplified. After trimming, segments with lengths of 460 bp were obtained and aligned with 16 16s rRNA sequence data from GenBank to find the best model. Accession numbers of downloaded sequences are provided in Suppl. material 3: Table S3. The best models of the phylogenetic trees are provided in Tables 2 and 3. The 16S rRNA sequence of *Cypraeagracilis* was selected as the outgroup.

There are some differences between the two trees. As shown in Bayesian tree (Suppl. material 9: Fig. S3), *Calpurnusverrucosus* is the sister group to *Crenavolvatraillii*, but the ML tree (Suppl. material 10: Fig. S4) shows that *Naviculavolvadeflexa* is the sister group of *Crenavolvatraillii*, and then the two groups jointly compose the sister group to *Calpurnusverrucosus* and *Primovulaformosa*. Despite these differences, the results of both showed that *S.bridgesi* and *S.triticea* were clustered in the same clade.

The greatest genetic distance between *S.triticea* and *S.bridgesi* was 0.0220% (MW411381 and OL589299). By contrast, the smallest interspecific genetic distance among the other specimens was 0.0860% (*Primovulaformosa* (OL589307) and *Crenavolvatraillii* (OL614740); *Primovulaformosa* (MW411392) and *Crenavolvatraillii* (KP033145). For more details, see Suppl. material 5: Table S5.

The amplified ITS sequences had lengths of 1200–1300 bp before trimming and approximately 1200 bp after trimming. Different clones (from the same individual) were highly similar, and the differences were concentrated in the SSR regions. In the high-quality part of the sequencing, the most common sequence of clones was selected. The ITS sequences used to build the tree were assembled by different clones (from the same individual). Results of BIC analysis showed that the best-fit models of ML tree and Bayesian tree are provided in Tables 2 and 3; the two types of phylogenetic trees were fully congruent. Suppl. material 11: Fig. S5 shows the phylogenetic tree combining support values of both models. The greatest and smallest genetic distances between *S.triticea* and *S.bridgesi* were 0.0077% (*Sandaliatriticea* (MW411406) and *Sandaliabridgesi* (MW411417)) and 0% (*Sandaliatriticea* (MW411407) and *Sandaliabridgesi* (MW411411), respectively, and the minimum interspecific genetic distance among the ITS sequences obtained in the present study was 0.1375% (*Primovulaformosa* (MW411419) and *Sandaliabridgesi* (MW411417)). For more details see Suppl. material 6: Table S6.

### ﻿Nomenclatural act

Based on morphological and molecular data, *Sandaliabridgesi* Lorenz, 2009 is here synonymised with *S.triticea* (Lamarck, 1810).

## ﻿Discussion

### ﻿Morphological data

The colouration of ovulids is variable, and many ovulid names have been introduced on basis of a few specimens; therefore, nominal species of Ovulidae often prove to be synonyms (Rosenberg 1992).

*Sandaliabridgesi* is the most recently described species in the genus *Sandalia* and was established based on morphological characters by Lorenz (2009). It was said to differ from *S.triticea* mainly in shell transparency and length-to-width ratio. However, in our collected specimens, we observed the presence of a continuous transition in the length-to-width ratios (Suppl. material 2: Table S2) and variations in transparency with observation angle, light intensity, and individual differences. Additionally, there is a co-evolution effect between Ovulidae and Gorgonacea (Reijnen 2016). Shell and mantle colour also show a high degree of variability due to influences by various environmental factors and therefore cannot be used as marker characteristics to distinguish between species (Rosenberg 1992; Schiaparelli et al. 2005). According to Rosenberg (1992), the colour pattern is a more reliable characteristic than colour per se. For instance, *Diminovulaculmen* (Cate, 1973), *Serratovolvadondani* (Cate, 1964), and *Crenavolvastriatula* (G.B. Sowerby I, 1828) exhibit diverse shell colour changes (Hardy 2020b). In certain species, such as *Crenavolvaaureola* (Fehse, 2002), coloured bands on the shell cannot be regarded as stable traits (Hardy 2020c). During our process of species identification, both *S.bridgesi* and *S.triticea* specimens were irradiated with UV light with wavelengths of 395, 365, and 254 nm, but the yellow fluorescence reported by Lorenz (2009) could not be observed. Therefore, fluorescence may not serve as a stable trait in Ovulidae. Given the subjectivity and instability involved in morphological identification, the use of molecular data for taxonomic identification may be the most effective method for resolving these issues.

### ﻿Molecular data

COI barcoding has been widely applied in identifying species belonging to the class Gastropoda (Stothard and Rollinson 1997; Hou et al. 2013; Quintero-Galvis and Raquel-Castro 2013; Layton et al. 2014). Research evidence has shown that sometimes COI is more capable of reflecting geographical differences than shell characters in certain taxa (Simison and Lindberg 1999). As an apparently rapidly evolving family of gastropod (Lorenz pers. comm. 7 July 2020; pers. obs.), Ovulidae have high phenotypic plasticity (Rosenberg 1992; Sánchez et al. 2016; Reijnen and van der Meij 2017; Lorenz 2020), leading to ambiguity in morphological classification. In recent years, researchers have utilised COI and 16S rRNA to investigate the phylogeny of Ovulidae and found that both are capable of distinguishing specimens at the species level (Schiaparelli et al. 2005; Sánchez et al. 2016; Reijnen and van der Meij 2019), resulting in the discovery of synonymy among ovulid species (Reijnen 2015). Meyer and Paulay (2005) utilised barcoding in the analysis of sequences of more than 2000 individuals in 263 taxa of the family Cypraeidae, the sister group to Ovulidae (Cate 1973; Rosenberg 1992; Meyer 2003, 2004), and found that identification of unknowns was 98% accurate with a neighbour-joining approach against an evolutionary significant unit (ESU) phylogeny. The correspondence between ESU definitions and traditional morphological taxonomy was high, with 255 ESUs (97%) recognised previously at either the specific or subspecific level, indicating that an ESU is a taxonomic unit equivalent to or smaller than a species. Therefore, traditional taxonomy within Cypraeidae at the species or subspecies level is supported by molecular data in addition to independent morphological criteria.

From the phylogenetic tree constructed using COI sequences (Suppl. material 8: Fig. S2), it can be observed that the sequences of *S.bridgesi* and *S.triticea* were clustered in the same clade, indicating the absence of significant genetic differentiation between the COI sequences of these specimens. Other clades were also well supported, which is in agreement with the findings of Meyer and Paulay (2005). The minimum interspecific genetic distance among the COI sequences of specimens other than *S.bridgesi* and *S.triticea* was approximately 5.7 times that of the maximum genetic distance between *S.bridgesi* and *S.triticea*, clearly demonstrating the high degree of similarity between the COI sequences of *S.bridgesi* and *S.triticea*.

The phylogenetic tree constructed from the 16S rRNA sequences showed that different specimens could be clearly distinguished at the species level using 16s rRNA (Suppl. materials 9, 10: Figs S3, S4). A study by Schiaparelli et al. (2005) showed that the minimum and maximum interspecific divergence values (obtained using the Jukes-Cantor model) of the 16S rRNA distance matrix between ovulid species were 0.03 and 22.3%, respectively. In the present study, the smallest genetic distance among species other than *S.triticea* and *S.bridgesi* was 0.0860%, supporting the findings reported by Schiaparelli et al. (2005). The greatest genetic distance between *S.triticea* and *S.bridgesi* was 0.0220%, which was approximately only a quarter of the smallest genetic distance among other specimens. Therefore, the 16S rRNA data further support the synonymy between *S.triticea* and *S.bridgesi*. Suppl. material 5: Table S5 illustrates the details of pairwise distance with the 16S sequences.

Being a non-transcribed spacer region, the ITS region is subject to smaller selective pressures and generally undergoes rapid evolution (Odorico and Miller 1997). It is commonly used for analysis at the population and species levels because of its high degree of sequence variation (Hillis and Dixon 1991; Harris and Crandall 2000). Therefore, ITS1-5.8S-ITS2 provides higher discriminating power at lower taxonomic levels. Among the ITS sequences obtained in the present study, the minimum interspecific genetic distance among specimens was approximately 18 times that of the greatest genetic distance between *S.triticea* and *S.bridgesi*, representing a significantly larger intraspecific-interspecific genetic distance ratio compared with COI and 16S rRNA. This indicates that genetic differentiation did not occur even in the rapidly evolving ITS1-5.8S-ITS2 gene region between *S.triticea* and *S.bridgesi*. In the ITS phylogenetic tree (Suppl. material 11: Fig. S5), *S.triticea* was convincingly clustered with *S.bridgesi* while the other clades were well supported.

## ﻿Conclusions

In conclusion, the COI, 16S rRNA, and ITS1-5.8S-ITS2 data of the ovulid specimens collected in the present study indicated the absence of genetic differences between *S.bridgesi* and *S.triticea*. Both the phylogenetic trees (Suppl. materials 8–11: Figs S2–S5) and pairwise distances (Suppl. materials 4–6: Tables S4–S6) show a high degree of similarity between *S.bridgesi* and *S.triticea*, suggesting that the morphological differences between the two species may be caused by phenotypic plasticity rather than genetic differences. Most ovulids are cryptic (Rosenberg 1992); the shell and mantle are usually imitating the colour pattern of their octocoral host. Therefore, the difference in colour pattern between the two species may be due to the different colours of the octocoral host.

This study indicated that the colour pattern might not be a reliable identification feature. We also compared the L/W ratio between the *S.meyeriana* holotype, *S.bridgesi*, and *S.triticea*, and there were no significant differences between them. According to Cate (1973), *S.meyeriana* (holotype: 19 mm) is larger than *S.triticea*, and the colour is white to pale violet (Lorenz and Fehse 2009). The front terminal tip of *S.meyeriana* is flat while that of *S.triticea* is sharp (pers. obs.). The taxonomic status of *S.meyeriana* needs further research.

As the high level of phenotypic plasticity in ovulid species results in much ambiguity in morphology-based classification criteria (Rosenberg 1992), the analysis of species through molecular approaches is of great significance to the elucidation of classification and evolutionary history (Schiaparelli et al. 2005). Based on the present knowledge, it is evident that striking phenomena of convergence and homoplasy characterise shell morphology in Ovulidae and that a molecular framework is necessary to recognise phylogenetically related groups.

## Supplementary materials

Supplementary material 1 Table S1 Data type occurences, Date, GenBank accession numbers Explanation note Specimens used in the analyses, including locality, date, and GenBank accession data. https://binary.pensoft.net/file/673866This dataset is made available under the Open Database License (http://opendatacommons.org/licenses/odbl/1.0/). The Open Database License (ODbL) is a license agreement intended to allow users to freely share, modify, and use this Dataset while maintaining this same freedom for others, provided that the original source and author(s) are credited.Qiong Wu

Supplementary material 2 Table S2 Data type morphological Explanation note L/W ratio of the genus *Sandalia* specimens. https://binary.pensoft.net/file/673867This dataset is made available under the Open Database License (http://opendatacommons.org/licenses/odbl/1.0/). The Open Database License (ODbL) is a license agreement intended to allow users to freely share, modify, and use this Dataset while maintaining this same freedom for others, provided that the original source and author(s) are credited.Qiong Wu

Supplementary material 3 Table S3 Data type GenBank accession numbers Explanation note Details of sequences obtained from GenBank. https://binary.pensoft.net/file/673868This dataset is made available under the Open Database License (http://opendatacommons.org/licenses/odbl/1.0/). The Open Database License (ODbL) is a license agreement intended to allow users to freely share, modify, and use this Dataset while maintaining this same freedom for others, provided that the original source and author(s) are credited.Qiong Wu

Supplementary material 4 Table S4 Data type phylogenetic Explanation note Pairwise genetic distance matrix of COI sequences among specimens of Ovulidae. The data of *S.triticea* specimens are marked in red. https://binary.pensoft.net/file/673869This dataset is made available under the Open Database License (http://opendatacommons.org/licenses/odbl/1.0/). The Open Database License (ODbL) is a license agreement intended to allow users to freely share, modify, and use this Dataset while maintaining this same freedom for others, provided that the original source and author(s) are credited.Qiong Wu

Supplementary material 5 Table S5 Data type phylogenetic Explanation note Pairwise genetic distance matrix of 16s sequences among specimens of Ovulidae. The data of *S.triticea* specimens are marked in red. https://binary.pensoft.net/file/673870This dataset is made available under the Open Database License (http://opendatacommons.org/licenses/odbl/1.0/). The Open Database License (ODbL) is a license agreement intended to allow users to freely share, modify, and use this Dataset while maintaining this same freedom for others, provided that the original source and author(s) are credited.Qiong Wu

Supplementary material 6 Table S6 Data type phylogenetic Explanation note Pairwise genetic distance matrix of ITS sequences among specimens of Ovulidae. The data of *S.triticea* specimens are marked in red. https://binary.pensoft.net/file/673871This dataset is made available under the Open Database License (http://opendatacommons.org/licenses/odbl/1.0/). The Open Database License (ODbL) is a license agreement intended to allow users to freely share, modify, and use this Dataset while maintaining this same freedom for others, provided that the original source and author(s) are credited.Qiong Wu

Supplementary material 7 Figure S1 Data type morphological Explanation note Dorsal, ventral, and lateral views of shells. A *Primovulaformosa* (G.B. Sowerby II, 1848) (20200722H2) B *Crenavolvatraillii* (A. Adams, 1856) (20210616BHH1) C *C.traillii* (20210616BHH9) D *Cuspivolvabellica* (Cate, 1973) (20210718H5) E *Phenacovolva* sp. (20210515DSH2). Scale bar: 2mm https://binary.pensoft.net/file/673872This dataset is made available under the Open Database License (http://opendatacommons.org/licenses/odbl/1.0/). The Open Database License (ODbL) is a license agreement intended to allow users to freely share, modify, and use this Dataset while maintaining this same freedom for others, provided that the original source and author(s) are credited.Qiong Wu

Supplementary material 8 Figure S2 Data type phylogenetic Explanation note ML phylogenomic tree constructed from COI sequences including *Sandaliabridgesi*, *S.triticea*, *Primovulaformosa*, *Naviculavolvadeflexa*, and other related species of the family Ovulidae. Bootstrap values are shown above the branch, while the support values based on MrBayes are shown in parentheses. The sequences of *S.triticea* are marked in red. *S.rhodia* is a synonym of *S.triticea* (Sartori 2014). https://binary.pensoft.net/file/673873This dataset is made available under the Open Database License (http://opendatacommons.org/licenses/odbl/1.0/). The Open Database License (ODbL) is a license agreement intended to allow users to freely share, modify, and use this Dataset while maintaining this same freedom for others, provided that the original source and author(s) are credited.Qiong Wu

Supplementary material 9 Figure S3 Data type phylogenetic Explanation note Bayesian phylogenomic tree constructed from 16S rRNA sequences including *Sandaliabridgesi*, *Sandaliatriticea*, *Primovulaformosa*, *Naviculavolvadeflexa*, and other related species of the family Ovulidae. The sequences of *S.triticea* specimens are marked in red. The coloured bands designate different species. https://binary.pensoft.net/file/673874This dataset is made available under the Open Database License (http://opendatacommons.org/licenses/odbl/1.0/). The Open Database License (ODbL) is a license agreement intended to allow users to freely share, modify, and use this Dataset while maintaining this same freedom for others, provided that the original source and author(s) are credited.Qiong Wu

Supplementary material 10 Figure S4 Data type phylogenetic Explanation note ML phylogenomic tree constructed from 16S rRNA sequences including *Sandaliabridgesi*, *S.triticea*, *Primovulaformosa*, *Naviculavolvadeflexa*, and other related species of the family Ovulidae. The sequences of *S.triticea* specimens are marked in red. The coloured bands designate different species. https://binary.pensoft.net/file/673875This dataset is made available under the Open Database License (http://opendatacommons.org/licenses/odbl/1.0/). The Open Database License (ODbL) is a license agreement intended to allow users to freely share, modify, and use this Dataset while maintaining this same freedom for others, provided that the original source and author(s) are credited.Qiong Wu

Supplementary material 11 Figure S5 Data type phylogenetic Explanation note ML phylogenomic tree constructed from ITS-region sequences including *S.bridgesi*, *S.triticea*, *Primovulaformosa*, and *Cuspivolvabellica*. Bootstrap values are shown above the branch, while the support values based on MrBayes are shown in parentheses. The sequences of *S.triticea* specimens are marked in red. https://binary.pensoft.net/file/673876This dataset is made available under the Open Database License (http://opendatacommons.org/licenses/odbl/1.0/). The Open Database License (ODbL) is a license agreement intended to allow users to freely share, modify, and use this Dataset while maintaining this same freedom for others, provided that the original source and author(s) are credited.Qiong Wu
