# Supplementary figures and images for: ﻿Molecular phylogeny suggests synonymy of Sandaliabridgesi Lorenz, 2009 with S.triticea (Lamarck, 1810) (Gastropoda, Ovulidae)
Source: Zookeys. 2022 Apr 18;1096:189–206. doi: 10.3897/zookeys.1096.79402 (PMC9038850; doi:10.3897/zookeys.1096.79402)

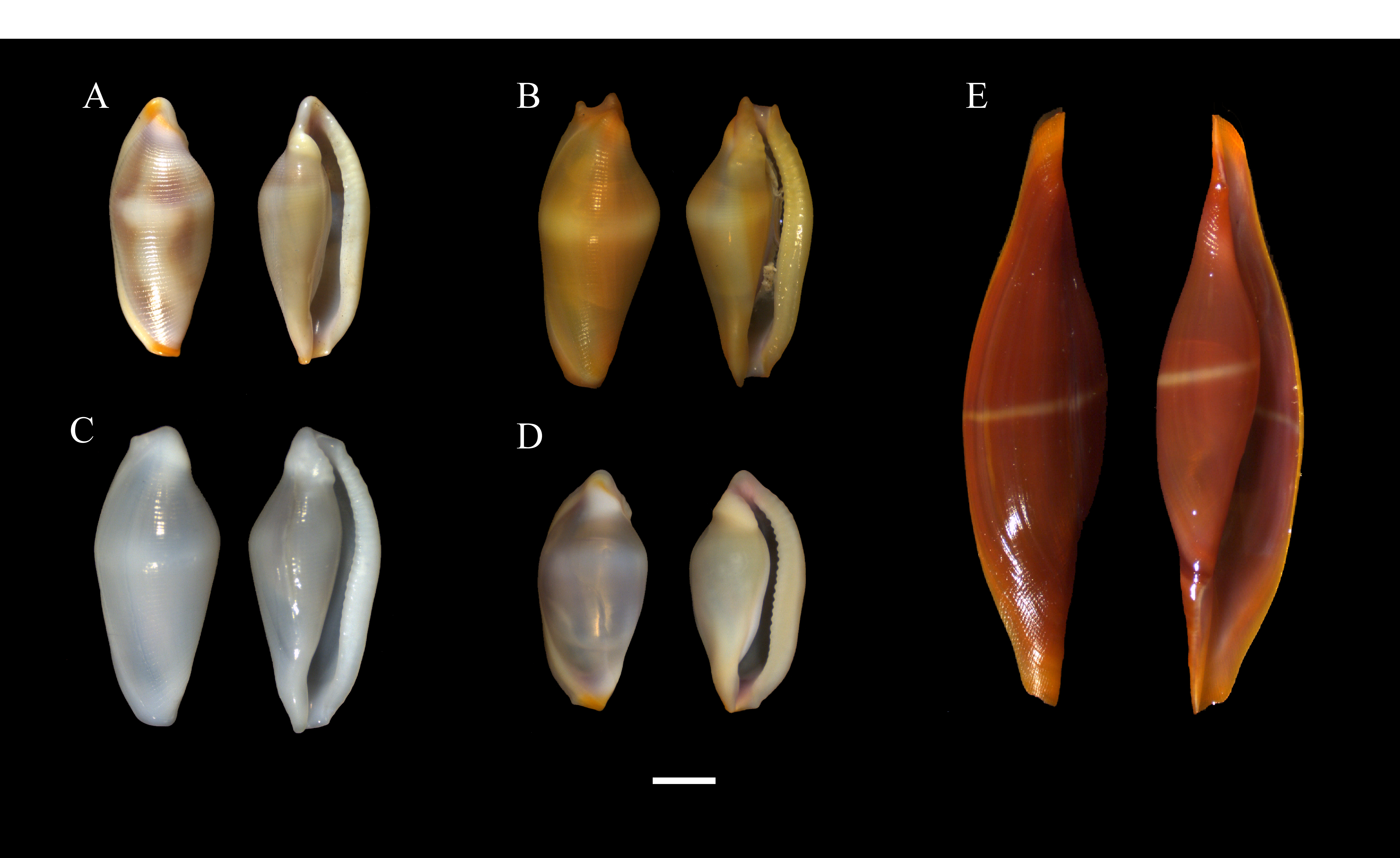

Supplement: Supplementary material 7 — Figure S1 [file zookeys-1096-189-s007.tif]
